# Argument and Verb Meaning Clustering From Expression Forms in LSE

**DOI:** 10.3389/fpsyg.2022.806526

**Published:** 2022-03-24

**Authors:** José M. García-Miguel, María del Carmen Cabeza-Pereiro

**Affiliations:** University of Vigo, Vigo, Spain

**Keywords:** valency, argument, micro-role, verb meaning, clustering, Spanish Sign Language

## Abstract

Languages use predicates and arguments to express events and event participants. In order to establish generalizations concerning the variety languages show regarding the strategies for discerning some arguments from the others, the concept of roles—and, particularly, macroroles, mesoroles, and microroles—associated with participants provides a widely studied starting point. In this article, the formal properties in the arguments of a set of 14 verb meanings in Spanish Sign Language have been analyzed. Arguments have been studied by considering their microroles, and a quantitative method for measuring distances from a plurality of properties has been adopted. The novelty of this analysis is that it focuses on how arguments group in terms of these properties. Subsequently, some generalizations justifying why some verb meanings have a tendency to associate with certain forms of argument expression are highlighted in this study.

## Introduction

This study deals with expression strategies used to distinguish participant roles in Spanish Sign Language (LSE) and has as its main goal to compare arguments of different verb meanings and to cluster them according to those strategies.

It is assumed that, in every language, it is possible to talk about events involving one or more participants. This is usually made by means of verbs used to predicate something about one or more arguments, i.e., people or things typically referred to by means of nouns. Argument structure, the set of constructions consisting of a predicate and the arguments depending on that predicate, is a core topic in (almost) every linguistic approach. However, relatively little research has been conducted on argument structure in sign languages. Each participant in a particular event performs a different role (microrole); for example, in an eating event, there is an “eater” microrole and a “food” microrole; in a breaking event, there can be a “breaker” and a “broken thing.” Microroles are, thus, event-specific; although they can be generalized across different event types at different levels of abstraction. For example, the “eater” and the “breaker” have in common that they can volitionally initiate the event; so they are both a so-called “Agent.” Labels of this kind are known as *semantic roles*, thematic roles, case roles (Fillmore, 1968), or mesoroles (Hartmann et al., 2014). At the higher level of generalization, some concepts, such as macroroles (Van Valin, 2004), proto-roles (Dowty, 1991), and the typological comparative concepts A, S, and P (Comrie, 1981; Haspelmath, 2011), have been formulated to capture the commonalities among different one-participant or two-participant events.

Languages differ in the coding strategies (morphosyntactic forms) used to distinguish participant roles in an event, but in general, these expression strategies can be classified into three main types (Haspelmath and Hartmann, 2015): (1) word order; (2) “flagging,” which subsumes case marking and adpositions; and (3) “indexing,” which subsumes person agreement or cross-referencing in the verb (cf. Haspelmath, 2013).

Signed languages, like any other language, also have strategies to refer to participants and their roles in an event, but the different modality (associated with the pervasive use of space, iconicity, and simultaneity) implies many relevant differences with spoken languages in the coding of argument structure (Geraci and Quer, 2014; Oomen, 2017; Kimmelman, 2022): “Flagging,” i.e., case-marking and adpositions, is (almost) absent in signed languages for the coding of core arguments. Word order is relevant in signed languages, but only a subset of arguments is referred to in discourse by means of an “independent” noun or pronoun sequentially placed before or after the verb. Many are left implicit (the referent being recoverable from context) or expressed somehow in simultaneity with the verb using indexing, classifiers, or role-shifting. Argument “indexing” (i.e., “agreement”) in spoken languages may have an analogous equivalent in many signed languages, which is known as agreeing or indicating verbs. These verbs have a path movement and use locations in the signing space so that the initial place of articulation matches the locus of an argument, whereas the final place aligns with the locus of a second argument. Some linguists analyze this as verbal agreement (Lillo-Martin and Meier, 2011) although some other researchers contest the validity of the parallel between spatial modification in sign languages and verbal agreement in spoken languages (Liddell, 2003; Schembri et al., 2018). We treat these phenomena as indexing and talk of “indicating” verbs. Classifier or depicting predicates are non-conventionalized (partly lexical) complex signs that include a handshape (a “classifier”) specifying a class of objects (e.g., an animate entity) and a movement depicting the sort of movement this entity performs in space (Supalla, 1986; Cuxac, 2000; Schembri, 2003; Morgan and Woll, 2007).^1^ Finally, facial expression and other non-manual components may be used in what is known as constructed action and role-shift, in which the signer is depicting the actions of a referent (Janzen, 2008; Cormier et al., 2013; Ferrara and Johnston, 2014); then, the role-shift becomes an additional device to refer to a participant in an event.

The morphosyntactic expression of arguments has served as the basis for the traditional classification of verbs in sign languages into three main types (Padden, 1988): agreement verbs, spatial verbs, and plain verbs. Other classifications (Oomen, 2020) distinguish between agreeing, classifier, body-anchored, and neutral verbs. Oomen (2018, 2020) has also developed the hypothesis that verb semantics impact on sign language verb type similarly to how it affects case-marking for transitivity in spoken languages (Malchukov, 2005) and subsequently she applies a semantic map for transitivity splits to German Sign Language (DGS) data. As an alternative approach, we propose an inductive quantitative method for the verb classification.

So far, we have been avoiding terms like ‘‘subject’’ and ‘‘object.’’ It is difficult to provide them with cross-linguistic valid definitions, but they are mainly used to refer to the first and second most prominent or core participants, as far as they can be identified in a given language by morphosyntactic properties, such as word order, case, or verbal agreement. However, the distribution of these properties is language-specific and leads to different alignment systems.^2^ As for signed languages, it is also difficult to find constant morphosyntactic properties that consistently identify grammatical relations such as “subject” and “object.” Agreeing or indicating verbs are only a subset of the verb inventory, and even with them, the path movement associated with core arguments (reportedly “subject” and “object”) is not always obligatory. Nevertheless, Meir et al. (2007) defend an association of the subject with the signer’s body (“body as subject”). Their research based on data from American Sign Language (ASL) and Israeli Sign Language (ISL) defends the hypothesis that, in sign languages, the body is generally associated with the single argument of mono-actant verbs and, in the case of bi- and tri-actant verbs, it is the agent argument, or the most similar to the agent, the one expressed with reference to the body (Meir et al., 2007, §. 4.1). If this is indeed the case, these arguments will tend to use expression strategies based on the signer’s body. In particular, indicating may point to the body even if it does not refer to the signer, and reference may be accompanied by constructed action and role-shifting.

Instead of taking semantic roles and grammatical relations as a starting point, Hartmann et al. (2014) proposed comparing languages at the level of the participant roles of individual verb meanings (microroles) and identifying semantic role clusters by studying cross-linguistic coexpression tendencies, i.e., the ways in which the individual microroles cluster with respect to their coding across a range of diverse languages. In particular languages, coding properties cluster arguments around specific regions of the semantic space, but “subject” and “object” are not used in that work as cross-linguistic categories. The valency patterns database ValPaL (Hartmann et al., 2013) is also built around verb meanings, microroles, and coding strategies in several dozens of languages.

In this study, we took microroles as the starting point and compared them by studying non-cross-linguistic but intra-linguistic coexpression tendencies in LSE. The main reason for doing so is the variability in discourse. None of the expression strategies (word order, indexing, role-shifting, etc.) used to refer to arguments is obligatory in continuous discourse, not even for verbs and arguments of a particular type. Consequently, we proposed analyzing the discourse distribution of expression strategies for individual arguments. For a selected set of 14 verb meanings, we observed, in an LSE corpus, the expression strategies used for each argument and then identified semantic role clusters of microroles that tend to be coexpressed, i.e., that are prone to be expressed using similar strategies: independent noun, preposed or postposed to the verb, use of role shifting, classifiers, and indexing.

Our analysis and methods are inspired by the concept of “Behavioral Profile” (Divjak and Gries, 2006, 2009; Gries, 2010), a corpus-based quantitative approach to semantics, which assumes a strong correlation between semantic and distributional properties. The method consists of coding every particular corpus instance of a linguistic unit (e.g., a verb meaning) in terms of morphosyntactic, syntactic, and semantic contextual characteristics. The resulting co-occurrence table is assessed by means of a hierarchical agglomerative cluster analysis so that units with a similar distribution are grouped together. The hypothesis is that similar distributions reflect similar meanings. Our method is different in that we analyzed and clustered not only lexical meanings (in our case, a set of verb meanings) but also the participant roles of the arguments of those verbs. In addition, the morphosyntactic features on which the cluster analysis will be based are the coding strategies used for every argument. The following sections present the data, methods, and results of this approach.

## Materials and Methods

This research has been conducted on an LSE corpus entirely recorded in the region of Vigo (Galicia, Spain). It consists of 24 video files analyzed with ELAN (ELAN (version 6.2), 2021). Its total length is almost 3 h 20 min (3:19:26 precisely), and it contains 7,570 tokens. These tokens have been analyzed as 2,777 clause-like units (CLUs), i.e., units that, as it happens with clauses traditionally identified with spoken languages, consist of a predicate and its arguments (Hodge and Johnston, 2014). This corpus includes 13 elicited narrations, 5 interviews, 1 episode of a web series recorded in Galicia with 4 deaf performers, and 2 files from other genres. Their distribution by length, tokens, and CLUs is specified in Table 1. Concerning the signing participants, there are 7 men and 4 women. Among them, 5 are in the age group from 55 to 69 years, and the other 6 are in the group from 40 to 54. All of them have been signing for at least 20 years, and the time of their first contact with LSE varies from their birth to when they were 17 years old (in 4 cases it was before they were 5, in 6 before 12, and in 1 after 12). The age of acquisition of the performers in the web series remains unknown.

**TABLE 1:** Corpus distribution by the type of discourse.

Types of discourse	Token no.	CLU no.
Narrations	2963	1375
Interviews	2596	897
Elicited examples	1546	306
Drama (web series)	377	163
Other	88	36
Total	7570	2777

Deaf signers and sign language (SL) interpreters (see “Acknowledgments”) have collaborated in the glossing process in ELAN, whereas the grammatical annotation has been carried out by the authors of this study. For every one of the 2,777 identified CLUs, the following have been annotated in different ELAN tiers:

1. ID-gloss. We follow the proposal of Johnston (2010) to consistently use the same capitalized word for all the occurrences of a sign, regardless of whether they are inflected or not. An ID-gloss, therefore, represents the lemma of a sign.
2. Categories for each lexical (noun, verb, adjective, etc.) or partly lexical unit. Partly lexical units are those that are not listed in a dictionary but are part of the signed discourse (classifiers, deictics, buoys, or gestures).^3^
3. Predicate arguments, which have been tagged as A1, A2, A3, etc., depending on the semantic structure of each verb, i.e., in terms of microroles.
4. The locus, when relevant, e.g., in indicating verbs.
5. The animacy of the referent, i.e., whether it is human, animate non-human, or inanimate.

From these annotations, it has been possible to study for every argument in every CLU the expression strategies, which are detailed below.

From the abovementioned sample, we have selected all those CLUs that include a token with one of these 14 verb meanings**: carry**, **explain**, **give**, **go**, **help**, **leave**, **look**, **say**, **search**, **sign**, **speak**, **take**, **think**, and **throw.^4^** This selection was conducted based on frequency criteria (at least 13 occurrences of that meaning in the corpus). However, some frequent verbs were excluded. In particular, the lexemes START and WANT were not included, since they occur mostly in combination with other verbs, forming verb series or periphrases. WAIT2 was also excluded due to its nature of discourse marker, which renders the analysis of its verbal character doubtful. The distribution of the selected verb meanings among the different types of discourse is specified in Table 2.

**TABLE 2:** Frequency of the selected verb meanings by discourse type.

Verb meaning	Drama	Elicited ex.	Interview	Narration	Other	Total
Carry	0	4	6	3	0	13
Explain	1	3	7	8	0	19
Give	0	14	4	12	0	30
Go	1	3	7	10	1	22
Help	1	9	0	10	0	20
Leave	0	1	0	39	0	40
Look	0	16	14	109	0	139
Say	1	7	11	3	7	29
Search	1	7	1	30	0	39
Sign	1	0	30	0	0	31
Speak	0	3	34	0	0	37
Take	3	12	0	77	0	92
Think	2	6	6	9	0	23
Throw	0	6	0	9	0	15
Total	11	91	120	319	8	549

The methodology of selecting the data by frequency has the consequence of ignoring some classes of verbs whose predicate is usually non-verbal, as it is the case of psychological verbs whose argument structure involves an Experimenter and a Theme, such as AFRAID, NERVOUS, and ANGRY (Oomen, 2017) or feeling verbs, such as FEEL-COLD or BE-ANGRY, which are built with a single participant (Oomen, 2018, 2020). Attributive, existential, or possessive constructions are also left out, with peculiar characteristics in sign languages (Herrero and Salazar, 2003; Zeshan and Perniss, 2008).

**FIGURE 1:**
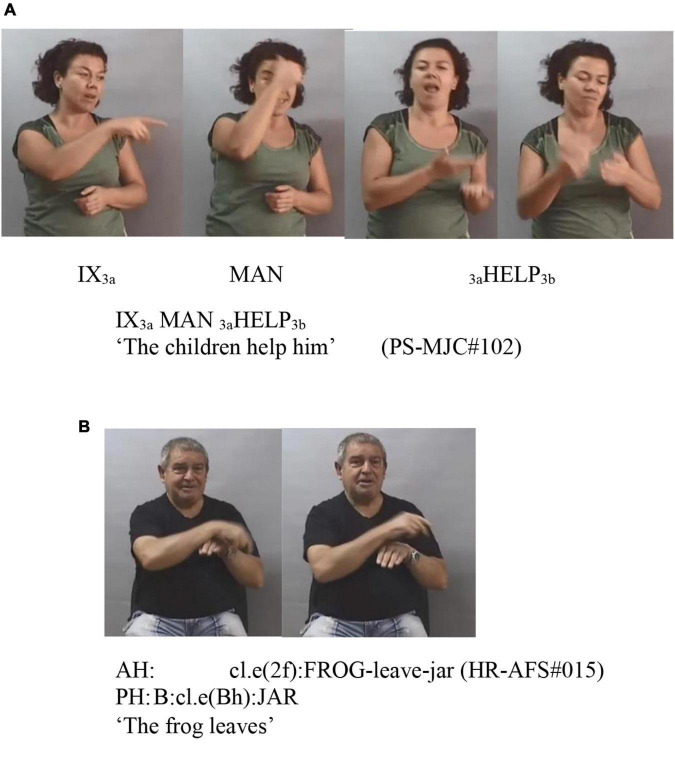
Examples **(A,B)**.

These verb meanings can be expressed through a single lexical form, as is the case with **help,** which is conveyed in the sign HELP. Nevertheless, in some cases, they are linked to two lexemes, such as the meaning of **go**, which has two lexemes: GO and GO2. We also have examples of a same meaning materializing through a lexical form (LEAVE) or through a classifying predicate (partly lexical form): *leave*. See Figure 1 for examples of a lexical (A) and partly lexical (B) forms. Undoubtedly, the fact that a verbal meaning is expressed through a lexical or partly lexical unit (“transfer units” in Sallandre and García, 2019) has consequences in the distribution of its arguments, as seen in the analysis of the results. In particular, it is characteristic of descriptive constructions (partly lexical) that one of the arguments is incorporated in the classifier predicate. Note that the term “partly lexical” warns that these units are not conventional, which does not mean that they do not follow certain formative patterns.

Every verb meaning denotes a type of situation with one or more intervening participants, each of them with a specific role (microrole). In the following lines, these 14 verb meanings are presented, together with the participants involved in each event type. Microrole labels, but not numbers, are taken from ValPaL (Hartmann et al., 2013). A total of 34 different argument roles (microroles) has been observed:

• **Carry** A1: carrier—A2: carried thing—A3: carrying goal.
• **Explain** A1: explainer—A2: explained content—A3: explaining addressee.
• **Give** A1: giver—A2: gift—A3: giving recipient.
• **Go** A1: goer—A2: going goal.
• **Help** A1: helper—A2: helpee.
• **Leave** A1: leaver—A2: left place/person.
• **Look** A1: looker—A2: looked at entity—A3: looked at place.
• **Say** A1: sayer—A2: said content—A3: saying addressee.
• **Search** A1: searcher—A2: searched for thing—A3: search location.
• **Sign** A1: signer.
• **Speak** A1: speaker.
• **Take** A1: taker—A2: taken thing—A3: taking source.
• **Think** A1: thinker—A2: thought content.
• **Throw** A1: thrower—A2: thrown thing—A3: throwing goal.

The indexes A1, A2, and A3 are specific for each verb, and they are theoretically arbitrary. Therefore, there should not be anything in common between, for example, the second argument in **help** and that of **search.** Nevertheless, the indexes are motivated by the prominence and relative frequency of each argument, so that, for example, all the agents are A1 (but not all the A1 are necessarily agents). Thus, A1 is the first candidate to become a subject, whereas A2 and A3 are, in principle, candidates to become an object. However, in many languages, the subject and object syntactic functions are characterized by presenting relatively constant expression forms (order, case, agreement, etc.). As explained in the following lines, the expression forms of these arguments in LSE are quite variable. The aim of this study is to find common expression patterns between different verb arguments within our data on the assumption that their expression similarities are semantically motivated.

All the examples registered in the corpus—from the annotated files in ELAN—for these 14 verb meanings (a total of 549 CLUs) together with the properties of their arguments have been extracted. The data have been reorganized in a 1,096-row and a 10-column table, in which rows show the arguments of every CLU and columns their varying expression properties. Table 3 summarizes the frequency of verbs and arguments. The index numbers identifying each microrole are theoretically arbitrary.

**TABLE 3:** Frequency of verb meaning and arguments.

Verb meaning	N_CLU	A1	A2	A3	Total_args
Carry	13	13	13	10	36
Explain	19	19	15	18	52
Give	30	30	29	30	89
Go	22	22	19	0	41
Help	20	20	20	0	40
Leave	40	40	21	0	61
Look	139	139	117	22	278
Say	29	29	28	13	70
Search	39	39	32	7	78
Sign	31	31	0	0	31
Speak	37	37	0	0	37
Take	92	92	92	19	203
Think	23	23	12	0	35
Throw	15	15	15	15	45
Total	549	549	413	134	1,096

The 10 variables in the dataset are Verb_meaning, MicroRol, Genre [=type of discourse], Animacy, Independence, Order, Role-shift, Classifier, Indexation, and Indexation locus. The first two variables are the ones that are described and used as clustering criteria for the data; genre and animacy are control variables; and the rest of them describe the expression strategies for every argument.

Six variables have been studied:

1. “Independent expression (indep)”: it is observed if there is an overt expression of an argument. There are three possible values: *L* (lexical element or pronoun), *0* (implicit or incorporated in the verb), or *X* (undetermined [e.g., reported speech]).
2. “Position of the independent elements (order)”: depending on whether an argument is placed before or after the verb, with four values: *a* (anteposition), *p* (postposition), *s* (sandwich, between two verbs), and *NA* (non-applicable [=not independent]).
3. “Role-shift (R)”: there is constructed action, and it reproduces the actions, thoughts, or locutions by the referent of a specific argument. The values are *R* (Role-shift applicable to the argument referent) and *0* (no role-shift).
4. “Classifier (cl)”: the argument in question takes the form of a manual classifier, in the active hand or in the passive hand, with three values: *cld* (active hand classifier), *cli* (passive hand classifier), and *0* (no applicable classifier for this argument).
5. “Indexation (Idx)”: it marks the initial or final location of the agreement verbs, with four values: *l1* (initial locus of movement), *I2* (final locus of movement), *I1/I2* (initial and final locus [reciprocal]), and *0* (no indexation, no verbal inflexion for agreement).
6. “Indexation locus (ILocus)”: indexation is produced in the signer locus or in a distal location, with the values: *s* (proximal location [in the signer’s body]), *n* (distal location [not in the signer’s body]), and *NA* (non-applicable [no indexation]).

The value levels for each of these variables have been gathered in Table 4, together with their frequency.

**TABLE 4:** Analyzed descriptive variables and assigned values.

Variable	Values	Description	Frequency
Indep		Independent expression	
	L	Lexicon or independent pronoun	347
	0	Implicit or incorporated in the verb	702
	X	Undetermined (e.g., reported speech)	47
Order		Position of independent elements	
	a	Before the verb (anteposition)	250
	p	After the verb (postposition)	88
	s	Between verbs [sandwich constructions]	9
	NA	Non-applicable [=not independent]	749
R		Role-shift	
	R	Role-shift applicable to the argument referent	203
	0	No role-shift	893
Cl		Classifier	
	cld	Active hand classifier	117
	cli	Passive hand classifier	37
	0	No applicable classifier for this argument	942
Idx		Indexation	
	I1	Initial locus of movement	197
	I2	Final locus of movement	244
	I1/I2	Initial and final locus (reciprocal)	8
	0	No indexation	647
ILocus		Indexation locus	
	s	Proximal location (in the signer’s body)	151
	n	Distal location (not in the signer’s body)	298
	NA	Non-applicable [no indexation]	647

This dataset has been analyzed using the hierarchical agglomerative cluster (HAC) analysis. This is a family of methods used to identify and represent (dis)similarity relations between different items on the basis of the variables that characterize the items. In our case, we analyze first the (dis)similarities between the 34 argument roles and, after that, the (dis)similarities between the 14 verb meanings. In both cases, the relative frequencies of each expression strategy function as the variables used to build the similarity matrix. All computations were carried out using R (R Core Team, 2020) following the steps reported by Divjak and Gries (2006, 2009) and Levshina (2015, chap. 15) to analyze behavioral profiles.

## Results

The proportion of appearance for the descriptive variables (Table 4) has been calculated for every argument of every verb. There do not seem to be obligatory expression strategies. Some expression procedures are certainly never used with certain arguments, but it is extremely rare that any of them happens in 100% of the cases.

For each of the values of every descriptive variable, we can order the arguments from those using these expression strategies in (almost) every case to those never using them.

**FIGURE 2:**
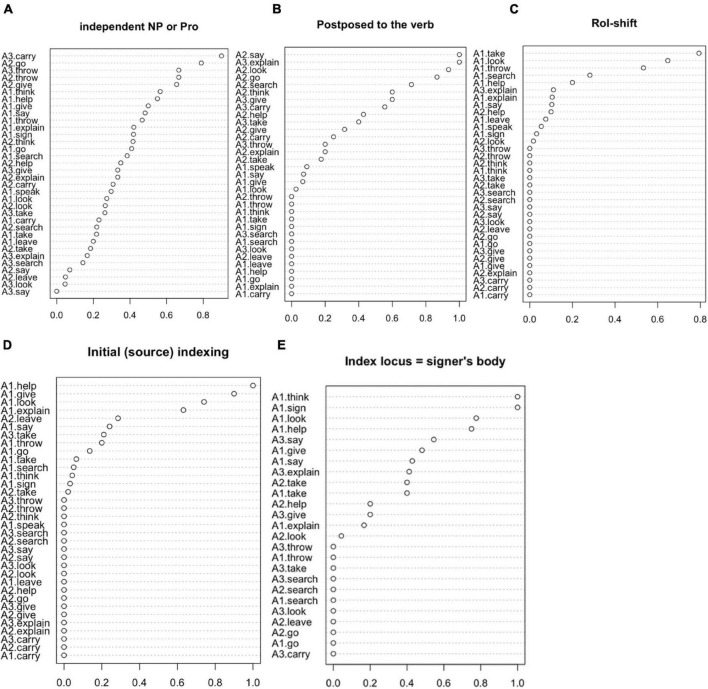
Proportion of arguments expressed according to descriptive values.

As an example, a particular argument can be overtly expressed—either through a lexical or an indexical form (pronoun). As it has already been stated in the previous section, the data come from different types of discourse. Therefore, most of the examples are contextualized. As a result, a frequent discursive elision of verb arguments has been detected. This can be observed in Figure 2A, where those with the highest frequency rate in being expressed independently are the A3 of **carry**, the A2 of **go**, the A3 and the A2 of **throw,** and the A2 of **give**, i.e., locations and manipulated objects. However, the most frequent scenario with the majority of the arguments is they are not overtly expressed, but deduced from context by the non-manual expression (role-shift) or expression procedures incorporated in the verb (classifiers and indexation).

### Computation of Distances and Clustering of Argument Roles

In order to calculate the “behavioral profile” for every argument, a co-occurrence table was construed by using the bp function of the RLing package (Levshina, 2015), which constructs behavioral profile vectors from categorical data, resulting in numeric vectors with concatenated proportions of each value in every variable in the data frame. Table 5 is a partial sample of behavioral profile vectors. For example, the argument *A1.carry* (“carrier”) is left implicit 77% of the times (indep.0 = 0.77) and lexically expressed 23% (indep.L = 0.23), and when lexically expressed, it is always preposed to the verb (order.a = 1.0).

**TABLE 5:** A partial sample of the table with behavioral profiles of arguments.

	indep.0	indep.L	order.a	order.p	order.s
A1.carry	0.77	0.23	1.00	0.00	0.00
A2.carry	0.69	0.31	0.50	0.25	0.25
A3.carry	0.00	0.90	0.44	0.56	0.00
A1.explain	0.58	0.42	1.00	0.00	0.00
A2.explain	0.40	0.33	0.80	0.20	0.00

*The numbers indicate proportions (between 0 and 1) of the given feature.*

If the values of two rows in the behavioral profiles table were identical, then we would say that the distance between arguments equals to 0. Otherwise, we can compute the distance between each pair of arguments. The more dissimilar their vectors, the greater their distances. The distance matrix is used to amalgamate the items exhibiting the highest similarity and successively amalgamate the resulting clusters until all clusters have been amalgamated. The resulting structure is typically represented by a dendrogram, i.e., a tree with all objects as leaves or branches.

**FIGURE 3:**
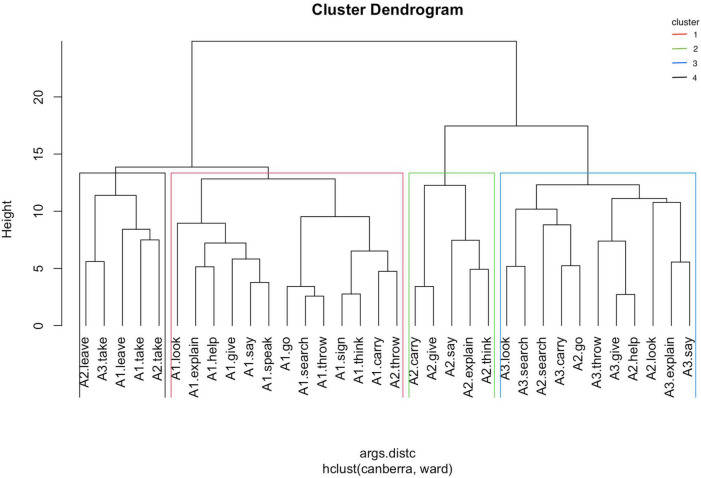
Cluster dendrogram of argument microroles.

The clustering algorithm is contingent on two important settings: (1) the measure of (dis)similarity and (2) the amalgamation strategy. Distances can be computed in R with the function dist() using different methods. We have chosen the “Canberra” method, which is more sensitive to differences between small values near zero. There are also different strategies of amalgamation, and we use the method “Ward.D2,” which usually produces compact and interpretable clusters (cf. Levshina, 2015, p. 306–312). Figure 3 depicts the clustering of verbs arguments in several hierarchical levels, representing the distances between the different items and clusters.

**FIGURE 4:**
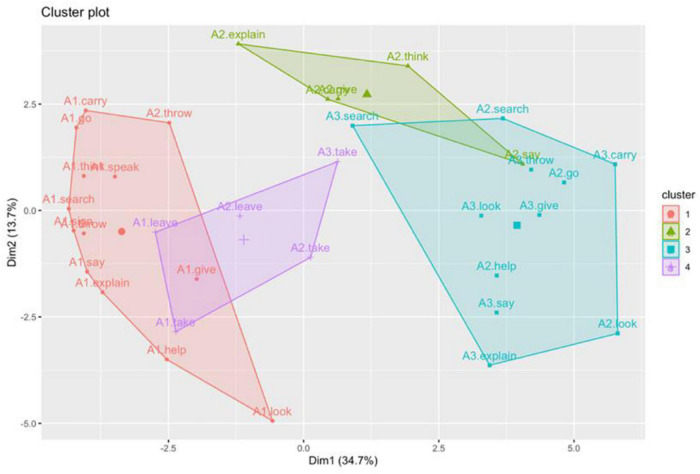
A scatterplot of the four argument clusters.

The optimal number of clusters can be determined by the so-called average silhouette width (ASW). However, in this case, such a method does not provide clear-cut results, the highest value (ASW = 0.23) corresponding to 15 clusters. This can be interpreted as an additional clue for the continuous nature of argument expression strategies. However, the observation of the dendrogram shows four relatively consistent clusters (ASW = 0.16). These four clusters have been highlighted in Figure 3. The relative distances between the members of the selected four clusters can be best perceived in a scatterplot (Figure 4), where intersections between groups 1 and 2, on the one hand, and groups 3 and 4, on the other hand, can be observed.

The following step in cluster analysis is to identify which of the variables drive the clustering, i.e., where the differences between clusters are. For each of these four groups, we calculate which properties show higher average proportions for every cluster in contrast with the others, and we select the features where the difference between proportions is the highest.

Cluster 1 consists of all the A1s (minus that of **leave**, which is in cluster 4). In addition, it includes the A2 of **throw**. These may be perceived as an anomaly, given the uniformity of the rest of the members of this cluster. The main features of these arguments are the following:

• Frequency of overt expression slightly above average (indep.L + 0.12)^5^ and, in this case, clear preference for anteposition (order.a + 0.45).
• Initial indexation (Idx.I1 + 0.28) localized in the signer’s body (ILocus.s + 0.31).
• Above-average use of role-shifting (R.R + 0.10).
• Use of classifiers less than average (cl.0 + 0.17).

Cluster 2 is composed by the A2s of **give**, **say**, **explain**, **carry,** and **think**. These are about given or carried objects and about what is said, explained, or thought. These are characterized by the following features:

• These arguments are usually not indexed (Idx.0 + 0.48).
• Indeterminate order: propositions and reported speech have been analyzed as independent clauses; hence, it is difficult to specify their order as arguments of a predicate (indep.X + 0.34), but when they are lexical elements, there is preference for the post-verbal position (order.p + 0.25).
• Slightly above-average use of classifiers (cl.cld + 0.09).

Cluster 3 consists of arguments sharing the trait of being the destination of a movement (A2 of **go**, A3s of **carry** and **throw**, and A3 of **search** and **look**) or the recipient of a transfer action (A3 of **give**, **say,** and **explain**; A2s of **look** and **help**). The A2 of **search** (an object) also belongs in this group. These are characterized by the following features:

• A clear association with final indexation (Idx.I2 + 0.80) with a preference for expressing locus different from the signer’s body (ILocus.n + 0.26).
• Preference for postposition (order.p + 0.39) when it is an independent expression.
• Less than average use of classifiers (cl.0 + 0.14) and role-shifting (R.0 + 0.10).

Cluster 4 gathers all the arguments of the verb meanings **leave** and **take**, which in LSE are (almost) always descriptive verbs.

• The arguments in these two verbs are expressed through classifiers (cl.d + 0.27, cl.i + 0.25, cl.0 −0.52).
• As a consequence, seldom are they expressed through lexicon (indep.0 + 0.29) or indexation (Idx.0 + 0.27).
• When they are lexically expressed, there is preference for the pre-verb position (order.a + 0.21).
• Usage of role-shifting slightly above average (R.R + 0.10), due to its use with A1 (but normally not with A2).

In the dendrogram in Figure 3, a hierarchically inferior level with a total of 7 clusters, subdividing three of the four main clusters, is perceived. Therefore, the so-tagged cluster 1 shows a separation between, on the one hand, the A1 of **look, help, explain, give, say,** and **speak** and, on the other hand, the A1 of **go, throw, search, think, sign,** and **carry**. At first glance, it does not seem like a consistent division.

Concerning the previous cluster 2, two types of objects it used to gather can be separated: the A2s of **give** and **carry**, on the one hand, and the A2 of **say**, **explain,** and **think**, on the other hand. These can refer to propositions or reported speech.

Regarding the previous cluster 3, there is a division, broadly speaking, between the A3 and the A2 referring to places and those acting as recipients of actions. In this case, the A3 of **throw** does not behave as expected, since it clusters with the recipients and not with the destinations.

### Computation of Distances and Clustering of Verb Meanings

**FIGURE 5:**
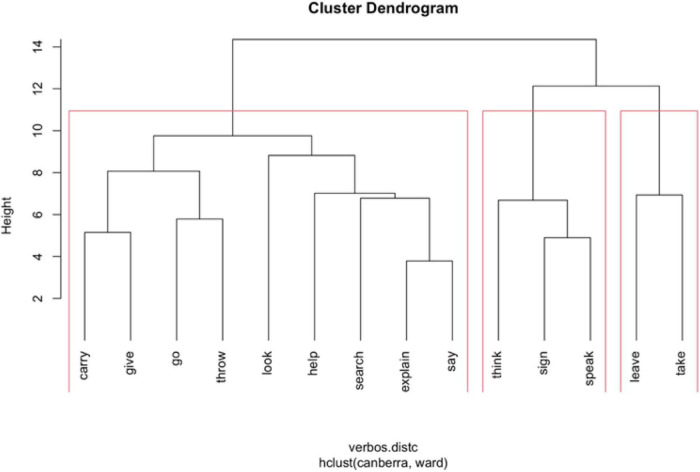
Cluster dendrogram of verb meanings.

**FIGURE 6:**
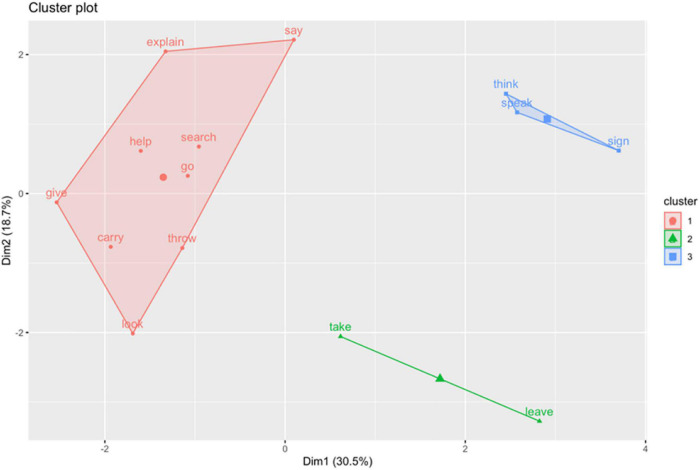
A scatterplot of the three verb meanings clusters.

The same method used to obtain the argument clusters has been subsequently applied to calculate the properties and clusters of verb meanings. Given that the arguments of every verb have different frequencies (see Table 3), the effect will be that the most frequent will be heavier in the verb’s profile and, therefore, A1 will be heavier in one-participant verb meanings than in three-participant ones. Figure 5 is the resulting dendrogram, where three groups have been highlighted for being the division with the highest ASW (0.202). The scatter plot of verb meanings shows clear distances between groups (Figure 6).

All the 9 verb meanings forming the first cluster (**give, carry, throw, go, look, help, search, say,** and **explain**) use space location procedures to express an argument that can equally represent a place (**carry, throw,** and **go**) or a recipient (**give, look, help**, **say,** and **explain**). The meaning of **search** always implies a place, regardless of what is being searched, an object or a person. Within this first group, there are two subgroups: one with **carry, give, throw,** and **go**, consisting of movement predicates (literal or metaphorical). These present the following traits more often than other groups of verbs:

• Indexation oriented toward locus not equal to signer (ILocus.n + 0.35).
• Compatible with lexical expression (indep.L + 0.22), frequently in the post-verbal position (order.p + 0.16).

The other subgroup with indicating predicates (**look, help, say,** and **explain**) shows the following features:

• Initial indexation (Idx.I1 + 0.17).
• There is no use of classifiers (cl.0 + 0.16).
• Relatively frequent use of role-shift (R.R + 0.07).

The second cluster is formed by **think**, **speak,** and **sign**. What they share is they are usually realized in mono-argumental constructions (100% of the cases with **speak** and **sign**; 47.8% with **think**). From the rest of verb meanings, only **leave** shows a similar percentage of mono-argumental use (47.5%). Since they are all typically monovalent verbs, the properties of their set of arguments are those of A1:

• Rare indexation (Idx.0 + 0.40), but when it occurs, it mostly comes from the signer (ILocus.s + 0.79).
• Rare role-shifting (R.0 + 0.09).
• Overt expression slightly higher than average (indep.L + 0.05), with a preference for anteposition (order.a + 0.19).

The third cluster consists of the verb meanings of **leave** and **take**, which is consistent with the results from argument clustering. Both are expressed through lexical (LEAVE) and descriptive (*leave*) predicates. Hence, their dominant properties are:

• Expression of arguments through classifiers (cl.cld + 0.38, cl.cli + 0.14).
• Seldom are they expressed through indexation (Idx.0 + 0.27) or independent expression (indep.0 + 029).
• Role-shifting (associated with argument A1) (R.R + 0.12).

## Discussion

This section aims to justify the obtained results, and in particular, to contextualize the hereby presented trends. It also intends to explain the apparent anomalous data as far as possible.

### Body as Subject

The arguments analyzed as A1 share the feature of referring to the most agentive participant of each verb or verbal meaning. Furthermore, in this sample, all of them are human (or, at least, animated). It can be, therefore, expected that most of them belong in the same group (Figure 3). The graph shows, however, a division between those A1s associated with meanings involving another participant and those which (mostly) do not indicate another argument or point a location. Among those from the first type, **talk** seems to be an anomaly, since **sign** belongs to the second group.

**FIGURE 7:**
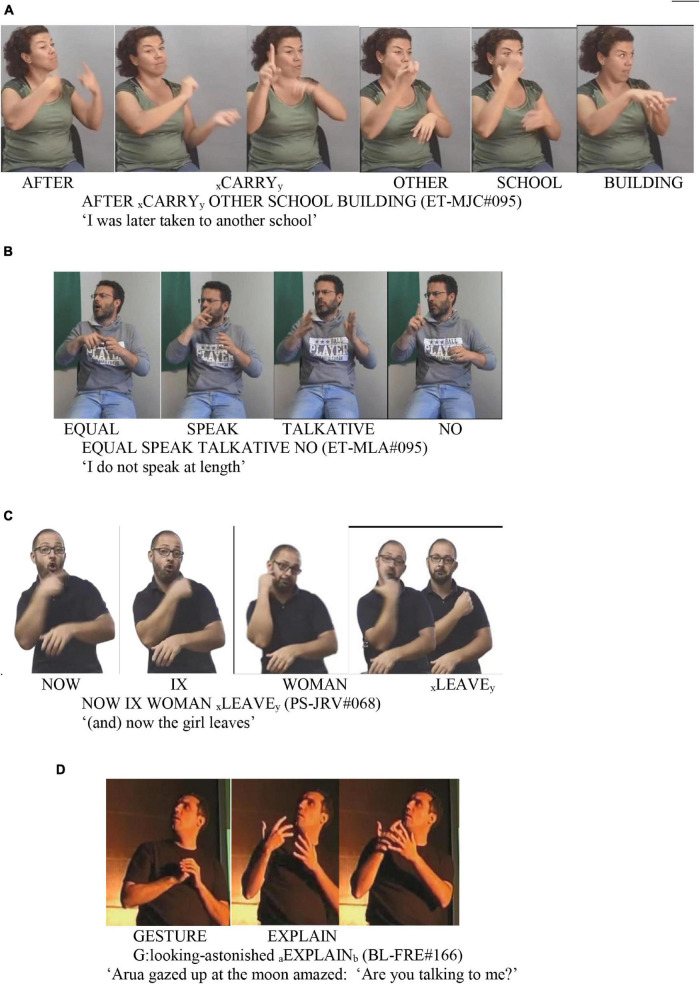
Examples **(A–D)**.

These results should be contrasted with the thesis by Meir et al. (2007) according to which the subject is identified with the body (“body as subject”). As it has been explained in Section 1, the authors defend the hypothesis that, in sign languages, the body is generally associated with the single argument of mono-actantial verbs and, in the case of bi- and tri-actantial verbs, it is the agent argument, or the most similar to the agent, the one expressed with reference to the body (Meir et al., 2007, §4.1). Even if this article does not discuss how the subject is expressed in sign languages (or if subject is or not an appropriate category for visuogestural languages), it can be assumed that there is a similarity between there hereby called A1 and the usual trend of the subject to be the most agentive and prominent argument. At least, it would be convenient to ask ourselves whether there is a *de facto* relationship between the A1 argument and the signer’s body. The two following studied expression strategies allow the establishment of this relationship:

- The initial indexation parameter (i.e., initial locus for movement, see Table 4) identifies mostly A1s. Moreover, some of the highest values of these A1s coincide with those of the locus in the signer’s body. This is particularly perceivable with meanings expressed through agreement verbs (**look, help, give,** and **say)** and less clear with the A1s associated with **explain** and **take.** The A1s of **think** and **sign** are those showing the clearest relation with the body. Among the arguments which show the movement origin, the least are the A1s of **carry, speak,** and **leave.** In the case of the first one (**carry**), the movement of the verb in its lexical form indicates a trajectory from one place to another, so that the initial index does not coincide with the body and is not similar to the A1 (e.g., Figure 7A). Frequently (46%, 6 examples out of 13), the A1 is a non-specified human entity, which favors a location far from the body, precisely in order to point an agent-backgrounding (Barberà and Cabredo, 2017). Regarding the A1 of **speak**, in its most frequent form, it is articulated at the height of the mouth, so it does not need such an indexation (e.g., Figure 7B). The A1 of **leave** is frequently expressed (26 examples out of 40) as a classifier in the active hand (never in the passive hand) so that the body indicates the origin or starting point from the distancing point. The same occurs when **leave** is expressed through a lexical form: the body of the signer represents a place from which they leave, and it is therefore not an A1 (e.g., Figure 7C). See also Figure 2: D *Initial (source) indexing* and D *Index locus* = *signer’s body*.

- The use of the role-shift reinforces the association agent-signer, since, generally speaking, A1 is the argument taking the role. Moreover, the association of constructed action with arguments other than A1 allows interpreting the CLU with agent-backgrounding so that the non-A1 argument is in focus. Thus, in Figure 7D, the participant being explained (Arua, the main character in the story) has more prominence that the one explaining (the moon). The A3 of **explain** and the A2 of **help**, both animated arguments, are associated with role-shifting. There are no similar examples with the A3s of **give** and **say**, despite also being expressed through agreement. As expected, there are no examples of constructed action in the A3s of **look** or **carry**, which indicate a location (see Figure 2C: *Role-shift*).

As a result, the quantitative analysis through applied clustering to LSE seems to confirm the thesis that A1 prefers to identify itself with the body of the signer. Nevertheless, there is also the possibility that the body points toward a location **(leave**) or that the indexation starts in a different locus than the body, particularly in verb meanings implying object movement (**carry**).

Furthermore, both the resource of using the A1 for a locus not related to the body (as it is the case with **carry**) and the identification of a different argument from that of A1 with the role are procedures, which have been identified in the specific literature as being responsible for a loss of prominence by the subject (or, more generically, the most agentive and prominent argument) (Guitteny, 2006; Janzen, 2008; Villanueva, 2010; Barberà and Cabredo, 2017; Leeson and Saeed, 2020; Kimmelman, 2022).

### Other Arguments Different From A1

When observing other arguments, the fact that those being analyzed as A2 associate participants that seem to have a limited similarity must be highlighted. In particular, only the A2s from **give**, **carry, say, explain,** and **think** are together in Figure 3. Others appear in different clusters:

- With other arguments of the same verb meanings: A2s of **take** and **leave**. These verb meanings are a singular grouping, as it can be observed in the corresponding graph (Figure 5). They share the feature of allowing the expression through lexemes and classifying predicates. In the case of **leave,** the classifier of an entity corresponds to the person leaving (A1), and in the case of **take**, it is A2 the one expressed in a classifier. There are no similarities between the A2 of **take** (the taken object) and the A2 of **leave** (the place from where they leave).
- With A1s: A2 of **throw**, analyzed below.
- With A3s: A2s of **look, search, help,** and **go**. They all share in common that they make reference to the location of a transference or a trajectory.

Concerning the A3, it should be stressed that they express human recipients (the person being given, told or explained) or places (where something is being thrown or taken). The A3s of **look** and **search** also represent places and appear to be associated with what is being looked at or searched for (A2). Oomen (2020) studies the formal differences between a class of agreement verbs and another of spatial verbs with data from DGS. However, this configuration does not reflect the clusters hereby analyzed, since it considers GO or LEAVE as spatial verbs (in different groups in the research in this work), whereas THROW, TAKE, and SEE are in the agreement group. The difference with the methodology used in this study is that Oomen does not analyze the arguments as separate entities, but she studies the formal property of verbs (e.g., having or not a handling handshape) and constructions (the constituent order). The explanation we propose for the fact that both human entities and locations appear clustered together is that all of them refer to a transfer or movement scheme, in which iconicity is the base for the conceptualization of these processes.

The results for the A2 and the A3 of **throw** were already similar in those calculations not considering the animacy factor. The former appeared with the A1s, and the latter showed more affinities with recipients than with places. In both cases, it can be interpreted that they behave as if they were discursively more prominent than others with which they apparently have more in common (A2s of **carry** and **give**, on the one hand, and A3 of **carry**, on the other hand). The expression traits they share (and which could justify this interpretation) are their tendency to be expressed independently (in lexeme or pronoun forms) and the fact that they can appear as passive or dominated-hand classifiers (Table 6). The final indexation, however, shows contradictory results for the A2 and A3 arguments of **throw**.

**TABLE 6:** Comparison of A2 and A3 of throw.

	Express indep.	Elided	Classif passive hand	Not classif	Final indexation
A2 of throw	66%	33%	13%	87%	0%
A3 of throw	66%	33%	20%	80%	100%

The result of combining the forms of expression with the animacy produces a relocation of the A2 of **throw** with other arguments intuitively closer, such as the A2 of **carry** and **give**.

### Clustering of Verb Meanings: Some Trends

Extracting conclusions from our results and their potential correlation with morphological classes in predicates, such as those proposed by Padden (1988), was not expected given the limited number of studied verbs and verb meanings. However, there seem to be a trend toward associations between the observed clusters and some specific morphological traits.

Particularly, a singularity of the group formed by the meanings of **leave** and **take**, characterized by their high frequency in the expression through classifying predicates, was observed. In addition, **give** and **carry** admit classifiers, although not in the proportion of the former, so they are not attracted toward the same group. Concerning this, in an early stage of data observation, it seemed that there were formal indications to treat together all the verbs implying object manipulation (**take, give, carry,** and also **throw**), since their A2 show anteposition percentages equal to or above 50%. However, when treated with other properties, this trend was blurred.

The results also allow to strengthen the relevance of expression through indexing or agreement verbs, which are those making the most use of space grammatical resources, both for expressing recipients/receivers (**help**, **give**, and **explain**) and places pointed to (**go**, **throw**, and **take**). From the semantic scope, they generically refer to a transfer or movement scheme that is iconically based.

For the meanings of **think** (cognition) and **speak** and **sign** (language), a different group is created. We consider that, in this association, the valency factor bears a lot of weight, since **speak** and **sign** are clearly monovalent, and **think**, even if it admits an object, usually has a general use (to be thinking).

## Conclusion

A quantitative approach has been applied to a corpus that—even if limited in extension—has been intensively and profusely analyzed.

The arguments of 14 verb meanings have been tagged as A1, A2, and A3, depending on their microrole structure so that A1 is the most agent-like (e.g., the giver, in the case of a giving process), A2 is the second participant implied in the process (e.g., given object), and A3, when present, is the third (e.g., given person).

The formal properties have been analyzed for every argument of every example, in order to, then, identify similarities through a clustering method.

The results can be summarized as follows: (1) tendency of A1s to cluster together, which indicates that similarities in the form imply similar patterns in conceptualization; (2) diversity of A2s, in correlation with the diversity of objects or goals selected by the different verb meanings; (3) proximity between the A3s referring to people and signaling locations, as they share indexation procedures; and (4) singular behavior of two verb meanings that are frequently expressed through classifiers.

In spite of the reduced size of the corpus, this analysis allows supporting some theses on verb meanings and their coding in sign language. Hence, the tendency most agent-like arguments (A1) show to associate with the signer’s body (as it is perceived through indexation and role-shifting) has been confirmed. Furthermore, those arguments signaling a human recipient or a destination (A3) are preferentially associated with verb meanings materialized through indexical procedures (agreement or indexing verbs). The singularity of arguments performed through classifying predicates has also been confirmed, particularly with the behavior of those arguments linked to the meanings of **take** and **leave**.

The relevance of this study for the knowledge of sign languages is demonstrated as the analyses in this study converge with previous research in sign languages (the body as a subject, the functional similarity of the indexing procedures, regardless of whether recipients or places are indicated). As far as spoken languages are concerned, the divergence of the procedures used to distinguish arguments in the clause constitutes a drawback when it comes to establishing generalizations.

## Data Availability Statement

The raw data supporting the conclusions of this article will be made available by the authors in isignos.uvigo.gal, without undue reservation.

## Ethics Statement

Written informed consent was obtained from the individual(s) for the publication of any potentially identifiable images or data included in this article.

## Author Contributions

JMG-M designed and applied the statistical formulation and clustering method. JMG-M and MCC-P have interpreted the results and written the text. Both authors have participated in the grammatical analysis of the data constituting the base of this study.

## Conflict of Interest

The authors declare that the research was conducted in the absence of any commercial or financial relationships that could be construed as a potential conflict of interest.

## Publisher’s Note

All claims expressed in this article are solely those of the authors and do not necessarily represent those of their affiliated organizations, or those of the publisher, the editors and the reviewers. Any product that may be evaluated in this article, or claim that may be made by its manufacturer, is not guaranteed or endorsed by the publisher.

## Footnotes

1 Cuxac (2000) offers a very detailed description of these forms of signed discourse from the perspective of the semiogenesis of language. He calls them “structures of great iconicity.” This approach includes manual and non-manual articulators, with special attention to the function of the gaze as an iconizing resource. This semiogenesis theory situates the most iconic forms of sign languages in the focus of the attention.

2 The term alignment refers to coexpression patterns between core arguments, i.e., which arguments use the same argument markers (flag or indexes). For example, an ergative system coexpresses S and P and uses a different marker to signal A arguments (cf. Haspelmath, 2011).

3 The distinction between lexical and partly-lexical units is taken from Johnston (2010, p. 125). From the perspective of the semiogenesis of language (Cuxac, 2000), other authors prefer a distinction between lexical units and “transfer units” (cf. Sallandre and García, 2019).

4 Given that the common convention in the literature on signing languages is glossing signs or sign lemmas in upper-case letters, this article uses the boldface format to specify verb meanings. Those expressions referring to classifying predicates are written in lower-case non-boldface letters, as they are not lexemes. In the case of lexeme glosses, numbers have been used to tag different lemmas with the same meanings; if a meaning specification needs to be included, it has been added after a full stop. The annotation in the classifiers provides information on the type of classifier (cl.e, entity classifier), and the hand configuration is indicated in brackets (2f, 2 fingers; Bh, horizontal B hand). AH stands for “active hand” and PH for “passive hand.” See example B in Figure 1. Pertinent locations have been signaled with sub-indexes: 1 for the signing person; a and b for locus in different positions of the signing space.

5 These abbreviations correspond to variables and values described in Table 4. The figures convey the difference between the average proportions of the cluster minus the average proportions of the remainder clusters.

## Supplementary Material

Supplementary Data Sheet 1 ArgStr_dataset.csv argument microroles and variables analyzed for cluster analysis.
